# The Role of Thrombomodulin in Estrogen-Receptor-Positive Breast Cancer Progression, Metastasis, and Curcumin Sensitivity

**DOI:** 10.3390/biomedicines11051384

**Published:** 2023-05-07

**Authors:** Chien-Yu Huang, Po-Li Wei, G. M. Shazzad Hossain Prince, Uyanga Batzorig, Cheng-Chin Lee, Yu-Jia Chang, Chin-Sheng Hung

**Affiliations:** 1 School of Medicine, National Tsing Hua University, Hsinchu 300044, Taiwan; cyhuang@life.nthu.edu.tw; 2 Institute of Molecular and Cellular Biology, National Tsing Hua University, Hsinchu 300044, Taiwan; 3 Department of Pathology, Wan Fang Hospital, Taipei Medical University, Taipei 11696, Taiwan; r5424012@tmu.edu.tw; 4 Department of Surgery, School of Medicine, College of Medicine, Taipei Medical University, Taipei 11031, Taiwan; poliwei@tmu.edu.tw (P.-L.W.); princegmsh@tmu.edu.tw (G.M.S.H.P.); 5 Division of Colorectal Surgery, Department of Surgery, Taipei Medical University Hospital, Taipei Medical University, Taipei 11031, Taiwan; 6 Cancer Research Center and Translational Laboratory, Department of Medical Research, Taipei Medical University Hospital, Taipei Medical University, Taipei 11031, Taiwan; 7 Graduate Institute of Cancer Biology and Drug Discovery, Taipei Medical University, Taipei 11031, Taiwan; 8 Department of Dermatology, University of California, La Jolla, San Diego, CA 92093, USA; buyangaaa@yahoo.com; 9 Graduate Institute of Medical Sciences, College of Medicine, Taipei Medical University, Taipei 11031, Taiwan; kerwinpipi@gmail.com; 10 Graduate Institute of Clinical Medicine, School of Medicine, College of Medicine, Taipei Medical University, Taipei 11031, Taiwan; 11 Cell Physiology and Molecular Image Research Center, Wan Fang Hospital, Taipei Medical University, Taipei 11031, Taiwan; 12 Division of Breast Surgery, Department of Surgery, Taipei Medical University Hospital, Taipei 11031, Taiwan

**Keywords:** breast cancer, thrombomodulin, estrogen receptor, progression, migration, curcumin, resistant

## Abstract

Estrogen and estrogen receptors (ER) play a key role in breast cancer progression, which can be treated with endocrine therapy. Nevertheless, resistance to endocrine therapies is developed over time. The tumor expression of thrombomodulin (TM) is correlated with favorable prognosis in several types of cancer. However, this correlation has not yet been confirmed in ER-positive (ER^+^) breast cancer. This study aims to evaluate the role of TM in ER^+^ breast cancer. Firstly, we found that lower TM expression correlates to poor overall survival (OS) and relapse-free survival (RFS) rates in ER^+^ breast cancer patients through Kaplan–Meier survival analysis (*p* < 0.05). Silencing TM in MCF7 cells (TM-KD) increased cell proliferation, migration, and invasion ability. Additionally, TM-KD MCF7 cells showed higher sensitivity (IC_50_ 15 μM) to the anti-cancer agent curcumin than the scrambled control cells. Conversely, overexpression of TM (TM-over) in T47D cells leads to decreased cell proliferation, migration, and invasion ability. Furthermore, TM-over T47D cells showed more resistance (IC_50_ > 40 μM) to the curcumin treatment. The PI staining, DAPI, and tunnel assay also confirmed that the curcumin-induced apoptosis in TM-KD MCF7 cells was higher (90.34%) than in the scrambled control cells (48.54%). Finally, the expressions of drug-resistant genes (ABCC1, LRP1, MRP5, and MDR1) were determined by qPCR. We found that the relative mRNA expression levels of ABCC1, LRP1, and MDR1 genes after curcumin treatment were higher in scrambled control cells than in TM-KD cells. In conclusion, our results demonstrated that TM plays a suppressive role in the progression and metastasis of ER^+^ breast cancer, and it regulates curcumin sensitivity by interfering with ABCC1, LRP1, and MDR1 gene expression.

## 1. Introduction

Breast cancer is the most diagnosed malignancy in women and is responsible for the fourth cancer-leading death in Taiwan [1,2]. Breast cancer is initially classified by the presence or absence of hormone receptors (HMR), such as estrogen receptors (ER), progesterone receptors (PR), and human epidermal growth factor receptors (HER-2) [3,4]. The expression patterns of these receptors usually decide the clinical treatments of choice. Most patients (75%) diagnosed with metastatic breast cancer are HMR-positive [5]. Endocrine therapy remains the treatment of choice for patients with minimal metastasis without life-threatening symptoms, given its similar survival rates and fewer side effects compared to the other chemotherapeutic regimen [6]. The most frequently used medicines are estrogen blockers or selective estrogen receptor modulators (SERMs) such as tamoxifen and aromatase inhibitors such as letrozole, anastrozole, and exemestane for postmenopausal females [6,7,8]. More than 20% of early-stage breast cancer patients develop metastatic disease eventually [9].

Thrombomodulin (TM) is a 74 kDa transmembrane receptor abundantly expressed on the endothelium [10] with functional roles in coagulation, inflammation, and carcinogenesis [11]. The effects of TM activity in cancer correlate to its regulatory roles in anticoagulation, anti-inflammation, tissue adhesion, and proliferation [12,13,14,15,16]. Although initial studies demonstrated TM as an anticoagulant, recent studies have revealed that TM may play a different role in inflammatory processes and tumor progression [17,18,19]. Loss of TM expression was observed in bladder cancer cells compared to normal bladder cells [20]. TM-producing HCC showed low intrahepatic metastasis, tumor thrombus, and capsular invasion frequency. Patients with TM-positive hepatocellular carcinoma (HCC) also have a higher recurrence-free survival when compared to patients with TM-negative HCC [15,21,22]. Plasma levels of TM are found to increase with the progression of various cancers, such as pancreatic and lung cancer, in humans [22]. Neutrophil extracellular traps (NETs) around metastatic tumors were observed in pancreatic cancer specimens. Targeting NETs with TM in pancreatic cancer can improve surgical outcomes [23]. Therefore, an increase in TM expression seems to play an essential role in carcinogenesis.

Curcumin is naturally extracted from rhizomes of the plant *Curcuma longa* [24,25]. *Curcuma longa* has been traditionally regarded as a medicinal plant and provides beneficial effects to the human body mainly through its antioxidant and anti-inflammatory mechanisms [26]. The health benefits of curcumin have been demonstrated in several studies, including the improvement of metabolic syndrome, arthritis, anxiety, and hyperlipidemia, as well as the alleviation of exercise-induced muscle soreness [27]. In recent years, increasing research has demonstrated the therapeutic effects of curcumin against various diseases [28], including diabetes [29], Alzheimer’s disease [30], and ulcerative colitis [31]. Furthermore, curcumin has been reported to present efficacy against several cancer cells [32,33], and it also regulates multiple redox state enzymes, transcription factors, adhesion molecules, protein kinases, and inflammatory cytokines [34,35]. Curcumin inhibits HCC by inducing endoplasmic reticulum stress and mitochondrial dysfunction [36]. Curcumin exerts efficacy by modulating ER and HER2 pathways in breast cancer [37,38]. Additionally, we previously found that the anti-cancer effect of curcumin in prostate hormone-refractory prostate cancer cells is determined by the expression level of maspin [39]. Therefore, curcumin holds the potential to be used as a supplementary anti-cancer agent, depending on the patient’s profile [40]. However, the role of TM in curcumin treatment in ER^+^ breast cancer is still unknown.

Breast cancer patients often suffer from chemotherapeutic resistance as the cancer cell evolves to acquire several mechanisms to avoid the adverse effects of chemo-drugs [40]. Acquired resistance to chemotherapeutic drugs occurs when cancer cells are exposed to some anti-cancer agents, which triggers the upregulation of several critical multi-drug resistance (MDR) genes [41]. In this study, we aim to unravel the unknown function of TM in ER^+^ breast cancer progression. We also investigated how TM expression level could affect curcumin treatment efficacy by regulating potential multi-drug resistance genes in ER^+^ cells. This would shed light on the role of TM in chemoresistance.

## 2. Methods and Materials

### 2.1. Cell Culture and Chemicals

The human ER^+^ breast cancer cell lines MCF7 and T47D were purchased from ATCC and cultured in Dulbecco Modified Eagle Medium (Thermo Fisher Scientific Inc., Waltham, MA, USA) supplemented with 10% fetal bovine serum (FBS, Thermo Fisher Scientific Inc., Waltham, MA USA) as well as 1 penicillin-streptomycin. Following trypsinization, cells were passaged every 2–3 days and maintained at 37 °C in 5% CO_2_. Curcumin, RNase A, propidium iodide (PI), and RNAzol reagent were purchased from Sigma-Aldrich (St. Louis, MO, USA). RIPA buffer, DAPI, and cDNA Reverse Transcription kit were purchased from Thermo Fisher Scientific, Waltham, MA, USA. For determining IC_50_ dose of curcumin by SRB assay (Sulforhodamine B), cells were treated with 0, 10, 20, and 40 μM curcumin.

### 2.2. Public Breast Cancer Datasets Analysis

Survival analysis was performed with a gene expression database named Kaplan–Meier Plotter (https://kmplot.com/analysis/, accessed on 17 April 2023). The Kaplan–Meier plotter can assess the effects of 54k genes (mRNA, miRNA, protein) on survival in 21 cancer types, including breast (*n* = 7830), ovarian (*n* = 2190), lung (*n* = 3452), and gastric (*n* = 1440) cancer. This web-based tool runs survival analysis based on the databases such as GEO, EGA, and TCGA. The primary purpose of the tool is to run meta-analysis-based discovery and validation of survival biomarkers [42]. In the breast cancer gene-chip dataset (mRNA), the correlation analysis was performed with the following options: probe for THBD (203887_s_at); splitting patients by median; survival: overall survival/relapse-free survival; follow-up threshold: 120 months; and restrict analysis of ER-positive subtypes (ER status—IHC). A default option was chosen for all other settings.

### 2.3. Electroporation-Based Transfer of shRNA

The shRNA inserted into the pLKO.1 plasmid was purchased from the National RNAi Core Facility (Academia Sinica, Taiwan; http://rnai.genmed.sinica.edu.tw, accessed on 17 April 2023). The shRNA containing the sequence targeting TM (NM_000361): GCCGATGTCATTTCCTTGCTA was transfected into MCF7 by electroporation using the Neon Transfection System (Thermo Fisher Scientific) with 2 pulses for 30 ms and 1245 V each time. Later, MCF7 cells stably expressing shRNA targeting TM (MCF7 TM-KD) were generated by puromycin (Sigma-Aldrich: St. Louis, MO, USA) selection.

### 2.4. Transfection by Lipofectamine

In brief, the TM gene was cloned into pcDNA^™^3.1D/V5-His-TOPO^®^ (Invitrogen^™^, Waltham, MA, USA) plasmid. According to the manufacturer’s protocol, lipofectamine^™^ 3000 (Invitrogen^™^, Waltham, MA, USA) was used to transfect T47D cells to overexpress the TM gene. Once the cells reached 70% confluency, 8 μg TM plasmid was transfected using lipofectamine^™^ 3000, and following that, TM expression was determined by Western blot.

### 2.5. Cell Cycle Determination

The cells were treated with curcumin for 48 h and then washed twice with PBS and fixed with ice-cold 70% ethanol overnight at 4 °C. After washing twice with PBS, cells were centrifuged and re-suspended in PBS containing 100 μg/mL RNAse A, 10 μg/mL PI. After that, cells were incubated for 30 min at room temperature in the dark. DNA content was measured with a BD FACSCanto II Flow Cytometer (BD Biosciences, San Diego, CA, USA), and results were analyzed with FlowJo software (FlowJo LLC, Ashland, OR, USA).

### 2.6. TUNEL Assay

The terminal deoxynucleotidyl transferase-mediated deoxy-UTP (dUTP) nick end labeling (TUNEL) assays were performed to detect apoptosis of MCF7 cells (Promega Corporation, Madison, WI, USA) following the manufacturer’s instructions. Parallel positive and negative controls were assessed simultaneously. Briefly, after the cells were harvested, cells were fixed with 1% formaldehyde solution for 15 min on ice. After washing with PBS, the cells were permeabilized with ice-cold 70% ethanol at −20 °C for 4 h or stored in 70% ethanol at −20 °C for one week. The cells were washed with PBS and incubated in an Equilibration Buffer at room temperature for 5 min. After the cells were equilibrated, Nucleotide Mix and rTdT Enzyme were added and incubated at 37 °C for 1 h in the dark. Next, cells were washed with wash buffer containing 0.1% Triton X-100 and 0.5% BSA and incubated in PI solution containing DNase-free RNase A for 30 min in the dark. Finally, cells were analyzed by flow cytometry.

### 2.7. Western Blot Analysis

After lysis of the cells with RIPA buffer (Thermo Fisher Scientific, Waltham, MA, USA), total protein concentration was measured using the BCA assay kit (Pierce Biotechnology, Inc., Rockford, IL, USA). About 20 µg protein samples were resolved by SDS-PAGE and then transferred by electrophoresis onto the PVDF membrane. The membranes were blocked with PBST solution (0.1% Tween-20) containing 5% skim milk for 1 h and probed with primary antibodies TM (SC7096) (Santa Cruz Biotechnology, Santa Cruz, CA, USA), GAPDH (G9545) (Sigma-Aldrich, St. Louis, MO, USA) MCL-1(#5453), Bcl-2(#2872) and Bax (#2772) (Cell Signaling Technology, Danvers, MA, USA) overnight at 4 °C, respectively. The membranes were washed with PBST and incubated with the HRP-conjugated secondary antibody (1:5000, Sigma) at room temperature for 2 h. Then, immunoblotting was visualized with chemiluminescence (ECL; Thermo Fisher Scientific) and ChemiDoc Image System (Bio-rad, Hercules, CA, USA).

### 2.8. Transwell Assay

Transwell assay was used to assess the invasion and migration ability. Approximately 500 µL cell suspension (4 × 10^4^ MCF7 cells or 3 × 10^4^ T47D cells) in a serum-free medium was loaded into the upper chambers either for migration assay (uncoated membrane) or invasion assay (Corning Matrigel^®^-coated chamber; BD Falcon, New York, NY, USA). A complete culture medium (1000 μL) with 10% FBS was added into the lower chamber. After 24 h culture, the cells underneath the transwell membrane were fixed with MeOH, stained with 0.1% crystal violet, and counted under a microscope.

### 2.9. xCELLigence Real-Time Cellular Analysis (RTCA) and SRB Assay

Real-time cell proliferation was measured using the xCELLigence Real-Time Cell Analyzer (RTCA) DP instrument with an E-plate (Roche Applied Science, Penzberg, Germany). After running the background blank with 100 μL complete culture medium, the cells were seeded in wells, and the cell proliferation was continuously monitored as the relative rate of change (cell index) every 30 min. All data were recorded using RTCA software. For the SRB assay, 7000 T47D cells were seeded in a 96-well plate. After a specific treatment period, cells were fixed with 10% trichloroacetic acid (TCA) overnight at 4 °C. Then, cells were washed with 1% acetic acid and stained with 0.04% SRB dye for 10 min at room temperature. SRB dye was released by adding 10 mM Tris-base solution, and optical density was measured by a microplate reader at 515 nM.

### 2.10. DAPI Staining

The cells were fixed with 4% formaldehyde for 15 min and then stained using DAPI (Thermo Fisher Scientific, Waltham, MA, USA) at a concentration of 3 nM for 10 min and observed under the fluorescence microscope for alterations in the nuclei.

### 2.11. Reverse Transcription-Quantitative (RT-qPCR)

Total RNA was extracted from cells using RNAzol reagent (Thermo Fisher Scientific, Waltham, MA, USA) according to the manufacturer’s instructions. Synthesis of cDNA was carried out through a reverse transcription reaction using cDNA Reverse Transcription Kits (Thermo Fisher Scientific, Waltham, MA, USA) on an Applied Biosystems 2720 Thermal Cycler (Thermo Fisher Scientific, Waltham, MA, USA). The thermocycling conditions were as follows: 25 °C for 10 min, 37 °C for 120 min, and 85 °C for 5 min. Individual gene expression analysis was then carried out using the SYBR Green kit in ABI 7500 FAST^TM^ detection system (Applied Biosystems, Foster City, CA, USA). The PCR program was set as follows: 95 °C for 30 s, 95 °C for 10 s, 60 °C for 30 s, for 40 cycles. The primer sequences were as follows: ABCC1 (Forward: 5′-TGCTGCACCAGTACTTCCACAT-3′; Reversed: 5′CCCCAATGACAGCGGTCTT-3′), LRP1 (Forward: 5′CAACAGATCAACGACGATGG-3′; Reversed: 5′-GGGTGGCGTCAGAGAAGTAG-3′), MRP5 (Forward: 5′-CGACCCCCTCAGTGCCTTA-3′; Reversed: 5′-GATGTTTCCGGATAGCACTATTGA-3′), MDR1 (Forward: 5′-GGCTCCGATACATGGTTTTCC-3′; Reversed:5′-CAGTGGTGTTTTTAGGGTCATCAA-3′), and GAPDH (Forward:5′-CCTGTACGCCAACACAGTGC-3′; Reversed: 5′-ATACTCCTGCTTGCT GATCC-3). GAPDH served as the internal control for quantitation. Data were analyzed using the 2^−∆∆Ct^ method.

### 2.12. Statistical Analysis

All data were expressed as mean ± SD for triplicate experiments. Statistical significance was examined using a two-tailed Student’s *t*-test. Survival analysis was obtained through the Kaplan–Meier Plotter website. The two patient cohorts were subjected to comparison via a Kaplan–Meier survival plot, with the calculation of the hazard ratio along with its corresponding 95% confidence intervals and log-rank *p*-value. A value of *p* < 0.05 was considered statistically significant.

## 3. Results

### 3.1. Higher TM Reflects Better Outcome in Breast Cancer Patients

**Figure 1:**
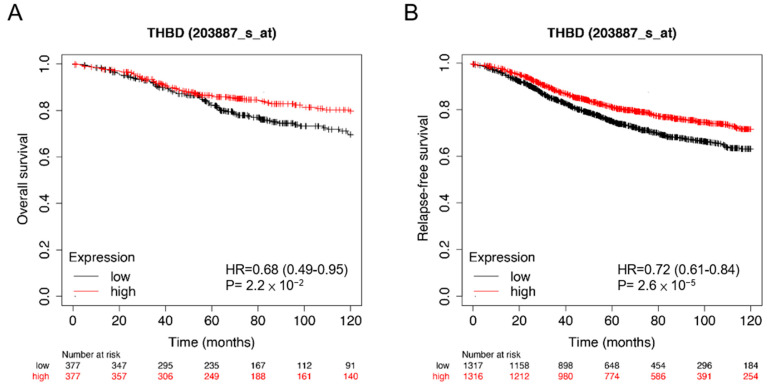
TM expression levels determines the clinical outcome in ER^+^ breast cancer patients. (**A**,**B**) Kaplan–Meier plots from ‘Kaplan–Meier plotter’ (https://kmplot.com/analysis/ accessed on 17 April 2023) show the correlation between TM expression and overall survival (**A**) or relapse-free survival in ER^+^ breast cancer patients (**B**). OS, overall survival; RFS, Relapse-free survival.

First, we examined the correlation between TM and the overall survival rate within ER^+^ breast cancer patients through Kaplan–Meier Plotter. As shown in Figure 1A, higher TM levels in patients (*n* = 377) showed a better overall survival rate compared to the lower TM in ER^+^ breast cancer patients (*n* = 377). In addition, we also determined the correlation of TM expression with the relapse-free survival rate and found that ER^+^ breast cancer patients with higher TM (*n* = 1316) had a better relapse-free survival rate compared to the lower TM in ER^+^ breast cancer patients (*n* = 1317) (Figure 1B). These data indicated that TM expression level could be a valuable biomarker of cancer prognosis and therapeutic response in ER^+^ breast cancer patients.

### 3.2. Silencing TM Promote Proliferation, Migration, and Invasion in MCF7 Cells

**Figure 2:**
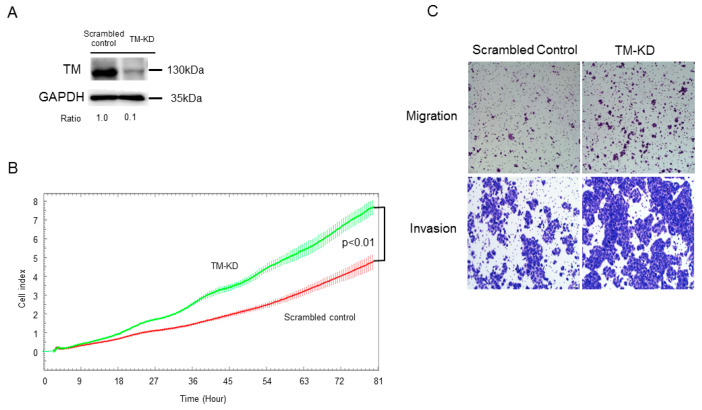
Silencing TM promoted malignant progression of ER^+^ breast cancer cell. (**A**) TM protein expression levels in scrambled control and TM-KD MCF7 cells were validated by Western blot. (**B**) Cell proliferation activity of scrambled control or TM-KD MCF7 cells was determined by the x’CELLigence system. (**C**) The migration and invasion abilities of scrambled control or TM-KD MCF7 cells were measured by transwell and invasion assays.

To further examine the role of TM in regulating breast cancer progression, TM knockdown cells were generated using the shRNA technique in MCF7 cells. As shown in Figure 2A, the knockdown efficiency of TM was over 80%, as indicated by qPCR and Western blot. We then determined the cell proliferation rate and found that silencing TM (TM-KD) increased cell proliferation ability in MCF7 cells (Figure 2B). Metastasis is the leading cause of cancer mortality. To assess whether TM is involved in cancer metastasis, cell invasion and migration ability of TM-KD and scrambled control MCF7 cells were investigated. As shown in Figure 2C, the knockdown of TM increased the number of migrated and invaded cells. Altogether, the data indicated that silenced TM contributed to an increase in cell proliferation, migration, and invasion in ER^+^ breast cancer cells.

### 3.3. Overexpression of TM Reduce Proliferation, Migration, and Invasion in T47D Cells

**Figure 3:**
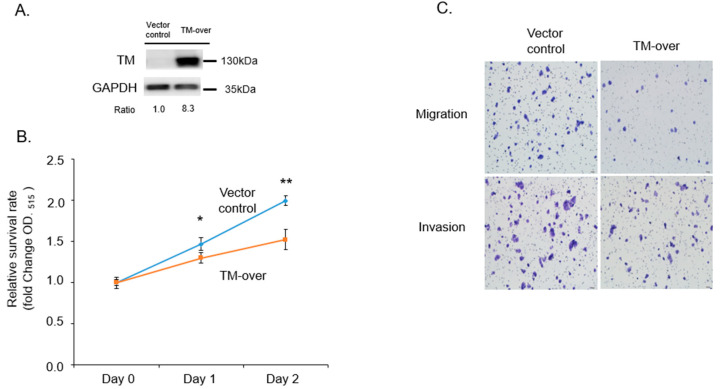
Overexpression of TM suppressed the malignant progression of ER^+^ breast cancer cell. (**A**) TM overexpression (TM-over) was determined by Western blot in T47D cells. (**B**) Vector control or TM-over T47D cells were seeded in a 96-well plate to determine the cell proliferation rate by SRB assay. (**C**) Vector control or TM-over T47D cells were seeded onto the trans-well migration or invasion chambers to determine the migration and invasion abilities. Data are presented as mean ± SD. * *p* < 0.05, ** *p* < 0.01.

We further overexpressed TM in T47D cells (Figure 3A) and verified proliferation, migration, and invasion activity. As shown in Figure 3B,C, overexpressed TM reduced proliferation, migration, and invasion activity in T47D cells. Those results indicate that TM plays a suppressive role in growth, migration, and invasion ability.

### 3.4. TM Mediates the Sensitivity of MCF7 and T47D Cells to Curcumin Treatment

**Figure 4:**
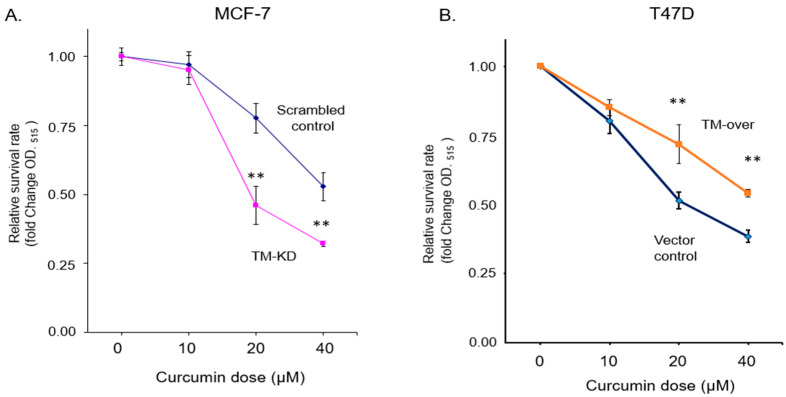
Silenced TM increased the sensitivity to the curcumin treatment in ER^+^ breast cancer cell. (**A**) Scrambled control or TM-KD MCF7 cells were treated with a range of curcumin concentrations as indicated for 48 h. The cell viability was determined by SRB assay. (**B**) Vector control and TM-over T47D cells were treated with curcumin for 48 h, and cell viability was determined by SRB assay. Data are presented as mean ± SD. ** *p* < 0.01.

Curcumin is a natural bioactive compound that has been demonstrated to have anti-cancer effects in several pre-clinical and clinical studies. To further explore the role of TM in drug response in ER^+^ breast cancer, we treated TM-KD and scrambled control MCF7 or TM-over and vector control T47D cells with different concentrations of curcumin for 48 h. The data showed that the cell viability decreased in TM-KD MCF7 cells in response to curcumin treatment. The IC_50_ of curcumin in scrambled control MCF7 was >20 µM, whereas 15 µM was observed in TM-KD cells (Figure 4A). Hence, we choose a 20 μM treatment dose for the following experiments in this study. The difference in the IC_50_ concentrations in response to the curcumin treatment demonstrated that the knockdown of TM-sensitized MCF7 cells to become highly susceptible to curcumin. In addition, TM-over T47D cells showed more resistance (IC_50_ > 40 µM) to curcumin exposure (Figure 4B). Together, TM mediates the curcumin response in ER+ breast cancer.

### 3.5. Knockdown of TM Alters Cell Cycle Distribution after Curcumin Treatment

**Figure 5:**
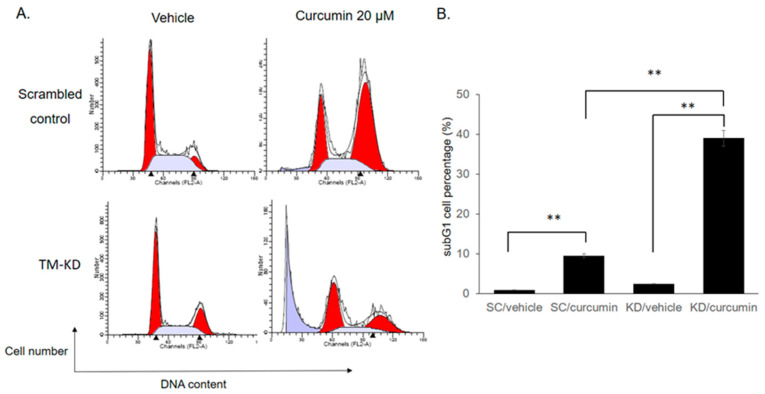

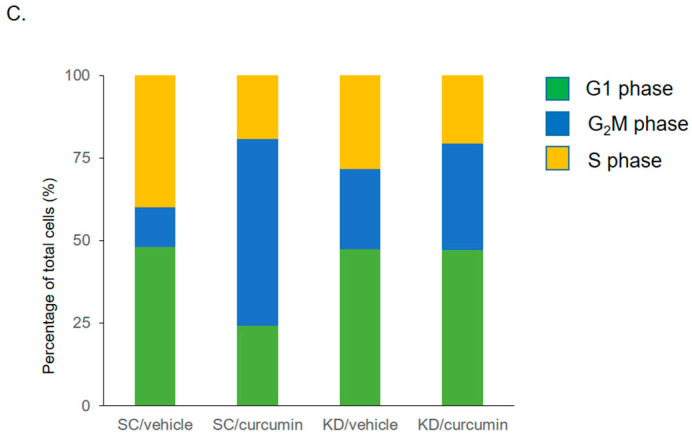
Knockdown of TM increased the cell number at the subG1 phase and decreased G2/M cell cycle arrest in MCF7 cells after curcumin treatment. (**A**) The cell cycle was analyzed by propidium iodide (PI) staining with flow cytometry following curcumin treatment (20 μM) for 48 h. (**B**,**C**) The quantified percentage of cells in the subG1 phase (**B**) and G1, S and G/2 M phases was plotted in a bar chart (**C**). Data are presented as mean ± SD. ** *p* < 0.01.

To investigate the effects of TM level on drug response, apoptotic cells in the sub-G1 phase were determined by propidium iodide (PI) flow cytometric assay. As shown in Figure 5, the knockdown of TM markedly increased the subG1 population following curcumin treatment. The subG1 population in scrambled control and TM-KD cells treated with curcumin (20 μM) was 9.45% and 39.06%, respectively. Interestingly, we noticed that the amount of G2/M accumulated cells was greater in scrambled control than in TM-KD cells after curcumin treatment. This implies that TM silencing possibly sensitizes MCF7 cells to undergo apoptosis rather than cell-cycle arrest once they are exposed to the curcumin.

### 3.6. TM-KD Cells Are More Susceptible to the Curcumin-Induced Apoptosis

**Figure 6:**
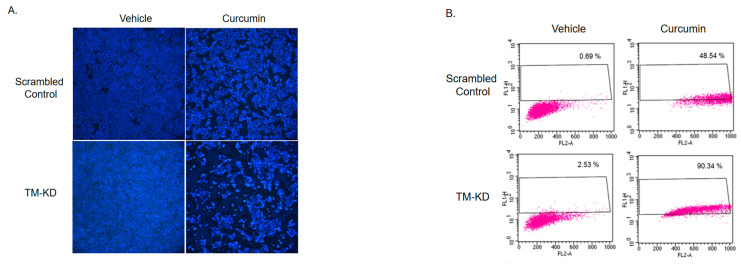

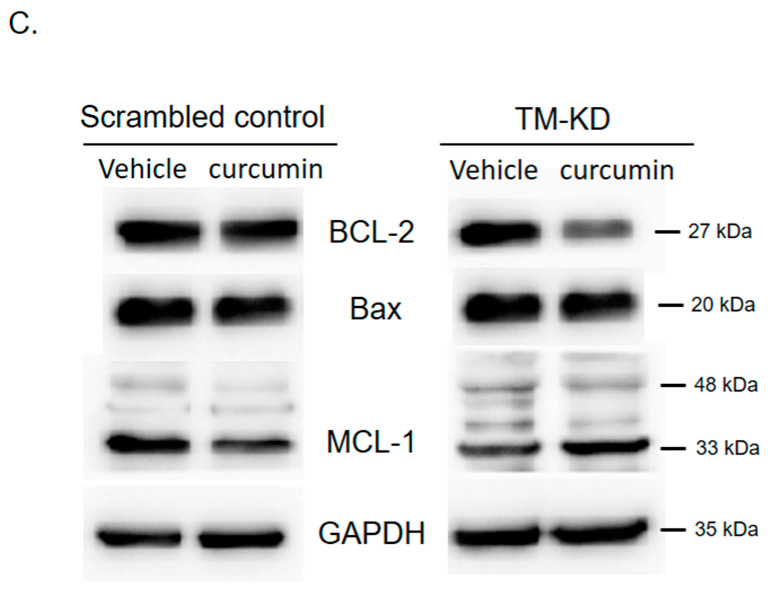
Knockdown of TM increased curcumin-induced apoptosis in MCF7 cells. (**A**) The cell density and chromatin condensation were examined by DAPI staining in scrambled control or TM-KD MCF7 cells. (**B**) Apoptosis was analyzed by flow cytometry using TUNEL assay after curcumin (20 μM) treatment for 48 h. (**C**) The expression of apoptosis-related proteins was assessed by Western blot.

To further examine the apoptotic effect of curcumin in scrambled control and TM-KD, morphological alterations of cell apoptosis were evaluated using DAPI staining, which detected the cell nucleus. Nuclear staining showed a lower cell density and greater extent of nuclear chromatin condensation in TM-KD cells in response to curcumin treatment. In addition, a TUNEL assay was also performed to detect and quantify apoptosis signals following curcumin treatment. As shown in Figure 6A, the apoptosis rate in TM-KD cells dramatically increased compared to the scrambled control cells after curcumin treatment. These results indicated that the knockdown TM affected the morphology of MCF7 cells and accelerated apoptosis in response to curcumin. In the TUNEL assay (Figure 6B), we also found more positive signals in TM-KD when exposed to curcumin (90.34%) compared to the scrambled control cells treated with curcumin (48.54%), indicating that TM level may influence sensitivity to curcumin. Further, we analyzed the expressions of the apoptosis-related molecules by Western blot. As shown in Figure 6C, we found that the Bcl-2 protein level was reduced in TM-KD cells treated with curcumin. The level of Bcl-2 remained unchanged in the vehicle and curcumin-exposed scrambled control cells. In addition, the level of Mcl-1 is reduced after curcumin exposure in scrambled control cells, but it was increased in TM-KD cells after curcumin treatment. Those results were consistent with our previous findings in this study.

### 3.7. Silencing TM Sensitizes MCF7 Cells to the Curcumin Treatment through Regulating Drug-Resistant Genes

**Figure 7:**
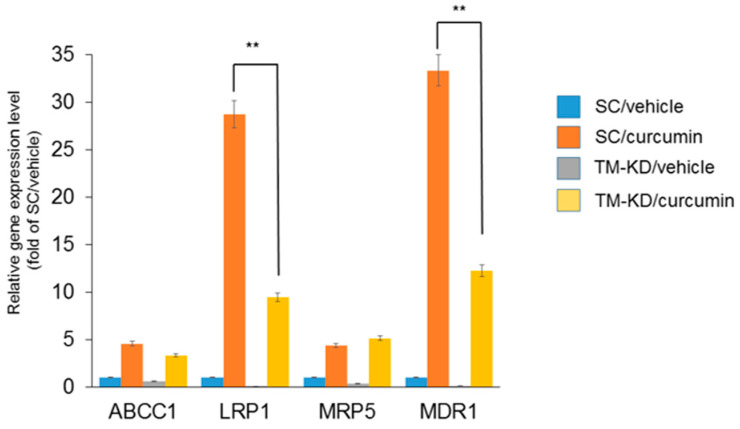
Silenced-TM reduced curcumin-induced upregulation of drug-resistant genes. The drug-resistant genes (ABCC1, LRP1, MRP5, and MDR1) levels in scrambled control and TM-KD MCF7 cells were determined by qPCR after curcumin treatment (20 μM). Data are presented as fold change over scrambled control/vehicle-treated samples (SC/vehicle). Data are presented as mean ± SD. ** *p* < 0.01.

In order to reveal the mechanism of TM in regulating curcumin sensitivity, expressions of drug-resistant genes (ABCC1, LRP1, MRP5, and MDR1) were determined by qPCR. As shown in Figure 7, we found that these gene expressions were lower in TM-KD cells than in the scrambled control. However, after curcumin exposure (20 μM), the induction folds of those MDR genes, especially LRP1 and MDR1, were higher in scrambled control cells than in TM-KD cells. This indicates an acquired drug resistance mechanism in ER^+^ breast cancer cells when exposed to the anti-cancer agent curcumin. However, silencing TM in MCF7 cells reversed this effect. These results imply that the knockdown of TM sensitizes MCF7 cells to curcumin by regulating drug-resistant genes.

## 4. Discussion

There is an urgent need to elucidate the possible candidate genes responsible for the progression and therapeutic responses in ER^+^ breast cancer patients. Our study revealed the role of TM in the progression, metastasis, and curcumin sensitivity in ER^+^ breast cancer. Moreover, lower TM expression is correlated to poor overall survival in ER^+^ patients. A previous study has reported that TM also regulates EMT and is related to doxorubicin resistance in lung cancer cells [43]. Reportedly, low TM expression is associated with increased expression of COX-2, which in turn increases fibronectin and vimentin levels, followed by EMT transformation in CRC cells [44]. We concluded that TM played a critical role in regulating ER^+^ breast cancer progression and sensitivity to the anti-cancer agent curcumin.

Curcumin has been demonstrated to be effective in cancer treatment with fewer side effects than traditional chemotherapy [26]. Numerous studies have demonstrated that curcumin causes cell cycle arrest at the G2/M phase [33,45,46]. Moreover, previous studies have reported that curcumin induces G2/M arrest and cyclin B1 change in pancreatic cancer cells [47]. Those results are consistent with our finding in ER^+^ breast cancer cell MCF7 that curcumin induced an increase in G2/M arrest. However, curcumin significantly induced the accumulation of more TM-KD cells at the subG1 phase than the scrambled control cells, indicating that silencing of TM facilitates an increased apoptosis rate upon exposure to curcumin. These results showed that the expression level of TM plays a crucial role in curcumin-induced cell cycle regulation in ER^+^ breast cancer cells. Although we propose that TM has a regulatory effect on the anticancer effects of curcumin, there are still issues with the bioavailability of curcumin that need to be addressed. Developing modified curcumins to reduce their metabolism rate and biological toxicity is a promising approach [48]. In addition, many researchers have been working on developing nanoparticle encapsulation techniques that can improve the bioavailability and delivery of curcumin [49]. Altogether, our findings can accelerate the application of curcumin to assist the existing therapeutic regimen for breast cancer.

Bcl-2 families belong to the apoptosis regulator-related proteins [50]. These proteins modulate mitochondrial outer membrane permeability and can be either pro-apoptotic (Bax) or anti-apoptotic (Bcl-2, MCL1) [36,51]. Our study found that curcumin treatment in MCF7 TM-KD cells decreased Bcl-2 level but increased MCL1 level, and the change in Bax protein level was marginal (Figure 5C). Interestingly, MCL1, an anti-apoptotic protein, can form a short-splicing variant with a pro-apoptotic role [52]. According to our results, MCL1 was increased after curcumin treatment in MCF7 TM-KD cells to exert its pro-apoptotic role. Curcumin combined with clinical radiation could be a more effective way of treating breast cancer patients according to TM expression [53]; however, further investigations are required.

Metastasis, a multi-step process involving several genes, dictates breast cancer treatment failure and mortality rate. Often, metastatic tumors are less responsive to chemotherapeutic drugs. [54]. Eventually, the tumor develops multidrug resistance, complicating the chemotherapeutic efficacy in patients [55]. Several mechanisms, some of which are acquired drug resistance, have been identified as an underlying cause of drug resistance [56,57]. To further clarify the relationship between TM expression level and curcumin resistance, we investigated the gene expression level of several drug-resistant genes such as LRP1, MDR1, ABCC1, and MRP5 after curcumin treatment in ER^+^ breast cancer cells.

The LRP1 gene encodes for the low-density lipoprotein receptor-related protein 1 (LRP1, also known as CD91), an endocytic and cell signaling receptor [58,59]. LRP1 mediates the endocytosis of a diverse set of extracellular ligands and is involved in multiple biological processes, including inflammation, proliferation, migration, apoptosis, and tumorigenesis [60]. LRP1 promotes cancer cell migration and invasion by regulating matrix metalloproteinase (MMP) expression and inhibits apoptosis by regulating the caspase and insulin receptor signaling pathway [61]. Furthermore, LRP1 modulates the ERK and JNK pathways to regulate focal adhesion and cancer cell invasion [62,63]. MDR1 (multidrug resistance protein 1) or P-glycoprotein 1 (Pgp), also known as ATP-binding cassette sub-family B member 1 (ABCB1), is a vital membrane ATP-dependent efflux pump [64]. It works as a salvage machine against harmful agents, such as medications and chemotherapeutics [65]. MDR1 overexpression is one of the primary mechanisms in reducing intracellular drug effects and developing multidrug resistance in cancers [66]. We found that curcumin treatment significantly increased LRP1 and MDR1 gene expression in scrambled control cells in comparison to TM-KD cells. The induction of gene expression in scramble control cells might be associated with an intrinsic drug resistance mechanism but silencing TM affects this. Hence, the TM expression profile could be a vital point for curcumin sensitivity, as TM expression regulates the expression of those MDR genes.

ABCC1 gene encodes for the protein MRP1 (multidrug resistance-associated protein 1), which belongs to the superfamilies of ATP-binding cassette transporters [67]. This protein works as an organic anion transporter, such as oxidizing glutathione, steroid hormones, and bile salts [68,69]. Accumulating evidence suggests that the higher expression of ABCC1 is related to drug resistance and poor survival of lung and breast cancer patients [70,71]. In prostate cancer, expression of ABCC1 was found to correlate with cancer stage and resistance [72,73]. The ABCC5 gene encodes MRP5 (multidrug resistance-associated protein 5). The ABC family plays a role in multidrug resistance in cancer treatment due to its ability to transport many drugs out of the cells [74,75]. Previous studies reported that MDR1 (ABCB1) and ABCC1 (MRP1) expressions in breast cancer are subtype-specific and associated with triple-negative breast cancer [76,77]. Medications such as ouabain may influence ATP-binding cassette (ABC) transporters and show different responses to chemotherapy in BC patients [77]. The cytotoxic effects of effective chemotherapeutic agents such as jadomycins are minimally affected by MDR1 (ABCB1), ABCC1 (MRP1), and ABCG2 efflux transporter function in MCF7 cells [78]. MRP5 (ABCC5) also confers resistance against pemetrexed in breast cancer [79]. MRP5 (ABCC5) expression also plays an essential role in osteoclast-mediated bone resorption, and it may be a predictable marker for bone metastasis in breast cancer [80]. Likewise, we found that silencing TM reduced the expression level of several MDR genes, which would allow ER^+^ MCF7 cells to become more susceptible to anti-cancer drugs.

“Cancer stem cells” may show intrinsic or “inherent” primary multidrug resistance [81,82]. Acquired or secondary multidrug resistance frequently occurs in tumors that developed and survived during pharmacotherapy [61,83]. According to our data, silencing TM expression would alter acquired drug resistance based on LRP1 and MDR1 expression levels upon curcumin treatment in ER^+^ breast cancer cells. However, ABCC1 and MRP5 expression were downregulated by TM silencing, but no significant change was observed based on the curcumin treatment. Together, this suggests a possible acquired resistance mechanism against curcumin based on TM expression. The underlying mechanism of curcumin sensitivity in TM-KD cells is related to the regulation of MDR genes, especially LRP1 and MDR1, by TM. This suggests that based on TM expression level, curcumin can be a treatment of choice in highly proliferative and metastatic cells. Curcumin has long been studied for its preventive role in cancer progression. Emerging studies have reported that human microbiota, especially gut microbiota, can regulate breast cancer progression [84,85,86]. Interestingly, curcumin holds the potential to modulate colon microbiota besides its anti-cancer effect [87,88,89]. Therefore, future studies could investigate whether curcumin can also modulate human microbiota in breast cancer patients with high or low TM expression levels and regulate cancer progression. This study has limitations, such as using in vitro system only. Although our study focused on ER^+^ breast cancer, the role of TM in more advanced triple-negative breast cancer (TNBC) could be studied in the future. Additionally, whether TM can regulate chemoresistance against existing therapeutic drugs other than curcumin for breast cancer treatment requires further investigation. Furthermore, investigations are required to dissect the acquired resistance mechanisms and to clarify TM as a potential biomarker of curcumin treatment in hormone-receptor-positive breast cancer.

## 5. Conclusions

In this study, we discovered the role of TM in ER^+^ breast cancer progression. Our bioinformatic analysis using an online database suggests that lower TM expression is correlated to poor outcomes in ER^+^ breast cancer patients. Likewise, we found that decreased TM expression in ER^+^ breast cancer cells increased cell proliferation, migration, and invasion ability, where overexpression of TM inhibited the malignant progression of ER+ breast cancer cells. Silencing TM expression enhanced the anti-cancer effect of curcumin, and in addition to that, TM expression levels influenced the modulation of ABCC1, LRP1, and MDR1 drug-resistant-related genes. Therefore, curcumin could be a promising, supplementary anti-cancer agent based on TM expression levels in ER^+^ breast cancer treatment. We concluded that TM could be a candidate biomarker for ER^+^ breast cancer progression and curcumin efficacy.

## Data Availability

The dataset supporting the conclusions of this article is included within the article.

## References

[1] Shen C.T., Chen F.M., Hsieh H.M. (2020). Effect of a national population-based breast cancer screening policy on participation in mammography and stage at breast cancer diagnosis in Taiwan. Health Policy.

[2] Youn H.J., Han W. (2020). A Review of the Epidemiology of Breast Cancer in Asia: Focus on Risk Factors. Asian Pac. J. Cancer Prev..

[3] Godone R.L.N., Leitao G.M., Araujo N.B., Castelletti C.H.M., Lima-Filho J.L., Martins D.B.G. (2018). Clinical and molecular aspects of breast cancer: Targets and therapies. Biomed. Pharmacother..

[4] Barzaman K., Karami J., Zarei Z., Hosseinzadeh A., Kazemi M.H., Moradi-Kalbolandi S., Safari E., Farahmand L. (2020). Breast cancer: Biology, biomarkers, and treatments. Int. Immunopharmacol..

[5] Wolff A.C., Hammond M.E., Hicks D.G., Dowsett M., McShane L.M., Allison K.H., Allred D.C., Bartlett J.M.S., Bilous M., Fritzgibbons P. (2014). Recommendations for human epidermal growth factor receptor 2 testing in breast cancer: American Society of Clinical Oncology/College of American Pathologists clinical practice guideline update. Arch. Pathol. Lab. Med..

[6] Mangini N.S., Wesolowski R., Ramaswamy B., Lustberg M.B., Berger M.J. (2015). Palbociclib: A Novel Cyclin-Dependent Kinase Inhibitor for Hormone Receptor-Positive Advanced Breast Cancer. Ann. Pharmacother..

[7] Chen X., Xu D., Li X., Zhang J., Xu W., Hou J., Zhang W., Tang J. (2019). Latest Overview of the Cyclin-Dependent Kinases 4/6 Inhibitors in Breast Cancer: The Past, the Present and the Future. J. Cancer..

[8] Reinert T., Barrios C.H. (2015). Optimal management of hormone receptor positive metastatic breast cancer in 2016. Ther. Adv. Med. Oncol..

[9] Cardoso F., Bischoff J., Brain E., Zotano A.G., Luck H.J., Tjan-Heijnen V.C., Tanner M., Aapro M. (2013). A review of the treatment of endocrine responsive metastatic breast cancer in postmenopausal women. Cancer Treat. Rev..

[10] Esmon C.T. (1987). The regulation of natural anticoagulant pathways. Science.

[11] Pindon A., Hantai D., Jandrot-Perrus M., Festoff B.W. (1997). Novel expression and localization of active thrombomodulin on the surface of mouse brain astrocytes. Glia.

[12] McCachren S.S., Diggs J., Weinberg J.B., Dittman W.A. (1991). Thrombomodulin expression by human blood monocytes and by human synovial tissue lining macrophages. Blood.

[13] Polster B.J., Westaway S.K., Nguyen T.M., Yoon M.Y., Hayflick S.J. (2010). Discordant expression of miR-103/7 and pantothenate kinase host genes in mouse. Mol. Genet. Metab..

[14] Kim S.J., Shiba E., Ishii H., Inoue T., Taguchi T., Tanji Y., Kimoto Y., Izukura M., Takai S. (1997). Thrombomodulin is a new biological and prognostic marker for breast cancer: An immunohistochemical study. Anticancer. Res..

[15] Lindahl A.K., Boffa M.C., Abildgaard U. (1993). Increased plasma thrombomodulin in cancer patients. Thromb. Haemost..

[16] Pang M., Zhao F., Yu P., Zhang X., Xiao H., Qiang W., Zhu H., Zhao L. (2021). The significance of coagulation and fibrinolysis-related parameters in predicting postoperative venous thrombosis in patients with breast cancer. Gland. Surg..

[17] Zhang Y., Weiler-Guettler H., Chen J., Wilhelm O., Deng Y., Qiu F., Nakagawa K., Klevesath M., Wilhelm S., Böhrer H. (1998). Thrombomodulin modulates growth of tumor cells independent of its anticoagulant activity. J. Clin. Invest..

[18] Horowitz N.A., Blevins E.A., Miller W.M., Perry A.R., Talmage K.E., Mullins E.S., Flick M.J., Queiroz K.C.S., Spek A., Conway E.M. (2011). Thrombomodulin is a determinant of metastasis through a mechanism linked to the thrombin binding domain but not the lectin-like domain. Blood.

[19] Kawamoto E., Nago N., Okamoto T., Gaowa A., Masui-Ito A., Akama Y., Darkwah S., Appiah M.G., Myint P.K., Obeng G. (2021). The Lectin-Like Domain of Thrombomodulin Inhibits beta1 Integrin-Dependent Binding of Human Breast Cancer-Derived Cell Lines to Fibronectin. Biomedicines.

[20] Ella-Tongwiis P., Lamb R.M., Makanga A., Shergill I., Hughes S.F. (2020). The role of antibody expression and their association with bladder cancer recurrence: A single-centre prospective clinical-pilot study in 35 patients. BMC Urol..

[21] Suehiro T., Shimada M., Matsumata T., Taketomi A., Yamamoto K., Sugimachi K. (1995). Thrombomodulin inhibits intrahepatic spread in human hepatocellular carcinoma. Hepatology.

[22] Ishii H., Majerus P.W. (1985). Thrombomodulin is present in human plasma and urine. J. Clin. Invest..

[23] Kajioka H., Kagawa S., Ito A., Yoshimoto M., Sakamoto S., Kikuchi S., Kuroda S., Yoshida R., Umeda Y., Noma K. (2021). Targeting neutrophil extracellular traps with thrombomodulin prevents pancreatic cancer metastasis. Cancer Lett..

[24] Sahebkar A. (2015). Dual effect of curcumin in preventing atherosclerosis: The potential role of pro-oxidant-antioxidant mechanisms. Nat. Prod. Res..

[25] Aggarwal B.B., Sundaram C., Malani N., Ichikawa H. (2007). Curcumin: The Indian solid gold. Adv. Exp. Med. Biol..

[26] Hewlings S.J., Kalman D.S. (2017). Curcumin: A Review of Its Effects on Human Health. Foods.

[27] Shah M., Murad W., Mubin S., Ullah O., Rehman N.U., Rahman M.H. (2022). Multiple health benefits of curcumin and its therapeutic potential. Environ. Sci. Pollut. Res. Int..

[28] Sultana S., Munir N., Mahmood Z., Riaz M., Akram M., Rebezov M., Kuderinova N., Moldabayeva Z., Shariati M.A., Rauf A. (2021). Molecular targets for the management of cancer using Curcuma longa Linn. phytoconstituents: A Review. Biomed. Pharmacother..

[29] Jiang Y., Qu K., Liu J., Wen Y., Duan B. (2022). Metabolomics study on liver of db/db mice treated with curcumin using UPLC-Q-TOF-MS. J. Pharm. Biomed. Anal..

[30] Tian M., Zhou F., Teng Z., Wang C., Zhang X., Wang Y., Li Y. (2021). Curcumin ameliorates lipid metabolic disorder and cognitive dysfunction via the ABCA1 transmembrane transport system in APP/PS1 double transgenic mice. J. Integr. Neurosci..

[31] Gupta S.C., Patchva S., Aggarwal B.B. (2013). Therapeutic roles of curcumin: Lessons learned from clinical trials. AAPS J..

[32] Bashang H., Tamma S. (2020). The use of curcumin as an effective adjuvant to cancer therapy: A short review. Biotechnol. Appl. Biochem..

[33] Liang H.H., Huang C.Y., Chou C.W., Makondi P.T., Huang M.T., Wei P.L., Chang Y.J. (2018). Heat shock protein 27 influences the anti-cancer effect of curcumin in colon cancer cells through ROS production and autophagy activation. Life Sci..

[34] Sahebkar A., Mohammadi A., Atabati A., Rahiman S., Tavallaie S., Iranshahi M., Akhlaghi S., Ferns G.A.A., Ghayour-Mobarhan M. (2013). Curcuminoids modulate pro-oxidant-antioxidant balance but not the immune response to heat shock protein 27 and oxidized LDL in obese individuals. Phytother. Res..

[35] Panahi Y., Alishiri G.H., Parvin S., Sahebkar A. (2016). Mitigation of Systemic Oxidative Stress by Curcuminoids in Osteoarthritis: Results of a Randomized Controlled Trial. J. Diet. Suppl..

[36] Liang H.H., Wei P.L., Hung C.S., Wu C.T., Wang W., Huang M.T., Chang Y.J. (2013). MicroRNA-200a/b influenced the therapeutic effects of curcumin in hepatocellular carcinoma (HCC) cells. Tumour Biol..

[37] Song X., Zhang M., Dai E., Luo Y. (2019). Molecular targets of curcumin in breast cancer (Review). Mol. Med. Rep..

[38] Wang Y., Yu J., Cui R., Lin J., Ding X. (2016). Curcumin in Treating Breast Cancer: A Review. J. Lab. Autom..

[39] Cheng W.L., Huang C.Y., Tai C.J., Chang Y.J., Hung C.S. (2018). Maspin Enhances the Anticancer Activity of Curcumin in Hormone-refractory Prostate Cancer Cells. Anticancer. Res..

[40] Pereira I.C., Mascarenhas I.F., Capetini V.C., Ferreira P.M.P., Rogero M.M., Torres-Leal F.L. (2022). Cellular reprogramming, chemoresistance, and dietary interventions in breast cancer. Crit. Rev. Oncol. Hematol..

[41] Emran T.B., Shahriar A., Mahmud A.R., Rahman T., Abir M.H., Siddiquee M.F., Ahmed H., Rahman N., Nainu F., Wahyudin E. (2022). Multidrug Resistance in Cancer: Understanding Molecular Mechanisms, Immunoprevention and Therapeutic Approaches. Front. Oncol..

[42] Gyorffy B. (2021). Survival analysis across the entire transcriptome identifies biomarkers with the highest prognostic power in breast cancer. Comput. Struct. Biotechnol. J..

[43] Yang Y., Cheng B.J., Lu S. (2017). Thrombomodulin regulates doxorubicin sensitivity through epithelial-mesenchymal transition in non-small cell lung cancer. Eur. Rev. Med. Pharmacol. Sci..

[44] Chang Y.J., Cheng Y.W., Lin R.K., Huang C.C., Chen W.T., Ke T.W., Wei P.L. (2016). Thrombomodulin Influences the Survival of Patients with Non-Metastatic Colorectal Cancer through Epithelial-To-Mesenchymal Transition (EMT). PLoS ONE.

[45] Li G., Wang Z., Chong T., Yang J., Li H., Chen H. (2017). Curcumin enhances the radiosensitivity of renal cancer cells by suppressing NF-kappaB signaling pathway. Biomed. Pharmacother..

[46] Wang Q., Fan H., Liu Y., Yin Z., Cai H., Liu J., Wang Z., Shao M., Sun X., Diao J. (2014). Curcumin enhances the radiosensitivity in nasopharyngeal carcinoma cells involving the reversal of differentially expressed long non-coding RNAs. Int. J. Oncol..

[47] Ling Y.H., el-Naggar A.K., Priebe W., Perez-Soler R. (1996). Cell cycle-dependent cytotoxicity, G2/M phase arrest, and disruption of p34cdc2/cyclin B1 activity induced by doxorubicin in synchronized P388 cells. Mol. Pharmacol..

[48] Flint A.L., Hansen D.W., Brown L.D., Stewart L.E., Ortiz E., Panda S.S. (2022). Modified Curcumins as Potential Drug Candidates for Breast Cancer: An Overview. Molecules.

[49] Tabanelli R., Brogi S., Calderone V. (2021). Improving Curcumin Bioavailability: Current Strategies and Future Perspectives. Pharmaceutics..

[50] Yu J., Zhou X., He X., Dai M., Zhang Q. (2011). Curcumin induces apoptosis involving bax/bcl-2 in human hepatoma SMMC-7721 cells. Asian Pac. J. Cancer Prev..

[51] Elyaman W., Terro F., Suen K.C., Yardin C., Chang R.C., Hugon J. (2002). BAD and Bcl-2 regulation are early events linking neuronal endoplasmic reticulum stress to mitochondria-mediated apoptosis. Brain Res. Mol. Brain Res..

[52] Bae J., Leo C.P., Hsu S.Y., Hsueh A.J. (2000). MCL-1S, a splicing variant of the antiapoptotic BCL-2 family member MCL-1, encodes a proapoptotic protein possessing only the BH3 domain. J. Biol. Chem..

[53] Minafra L., Porcino N., Bravata V., Gaglio D., Bonanomi M., Amore E., Cammarata F.P., Russo G., Militello C., Savoca G. (2019). Radiosensitizing effect of curcumin-loaded lipid nanoparticles in breast cancer cells. Sci. Rep..

[54] Patel L.R., Camacho D.F., Shiozawa Y., Pienta K.J., Taichman R.S. (2011). Mechanisms of cancer cell metastasis to the bone: A multistep process. Future Oncol..

[55] Choi Y.H., Yu A.M. (2014). ABC transporters in multidrug resistance and pharmacokinetics, and strategies for drug development. Curr. Pharm. Des..

[56] Higgins C.F. (2007). Multiple molecular mechanisms for multidrug resistance transporters. Nature.

[57] Tamaki A., Ierano C., Szakacs G., Robey R.W., Bates S.E. (2011). The controversial role of ABC transporters in clinical oncology. Essays Biochem..

[58] Kang H.S., Kim J., Lee H.J., Kwon B.M., Lee D.K., Hong S.H. (2014). LRP1-dependent pepsin clearance induced by 2’-hydroxycinnamaldehyde attenuates breast cancer cell invasion. Int. J. Biochem. Cell. Biol..

[59] Mantuano E., Lam M.S., Gonias S.L. (2013). LRP1 assembles unique co-receptor systems to initiate cell signaling in response to tissue-type plasminogen activator and myelin-associated glycoprotein. J. Biol. Chem..

[60] Kasza A., Petersen H.H., Heegaard C.W., Oka K., Christensen A., Dubin A., Chan L., Andreasen P.A. (1997). Specificity of serine proteinase/serpin complex binding to very-low-density lipoprotein receptor and alpha2-macroglobulin receptor/low-density-lipoprotein-receptor-related protein. Eur. J. Biochem..

[61] Xing P., Liao Z., Ren Z., Zhao J., Song F., Wang G., Chen K., Yang J. (2016). Roles of low-density lipoprotein receptor-related protein 1 in tumors. Chin. J. Cancer..

[62] Roura S., Cal R., Galvez-Monton C., Revuelta-Lopez E., Nasarre L., Badimon L., Bayes-Genis A., Llorente-Cortés V. (2014). Inverse relationship between raft LRP1 localization and non-raft ERK1,2/MMP9 activation in idiopathic dilated cardiomyopathy: Potential impact in ventricular remodeling. Int. J. Cardiol..

[63] Fuentealba R.A., Liu Q., Kanekiyo T., Zhang J., Bu G. (2009). Low density lipoprotein receptor-related protein 1 promotes anti-apoptotic signaling in neurons by activating Akt survival pathway. J. Biol. Chem..

[64] Wolking S., Schaeffeler E., Lerche H., Schwab M., Nies A.T. (2015). Impact of Genetic Polymorphisms of ABCB1 (MDR1, P-Glycoprotein) on Drug Disposition and Potential Clinical Implications: Update of the Literature. Clin. Pharmacokinet..

[65] Sui H., Fan Z.Z., Li Q. (2012). Signal transduction pathways and transcriptional mechanisms of ABCB1/Pgp-mediated multiple drug resistance in human cancer cells. J. Int. Med. Res..

[66] Breier A., Gibalova L., Seres M., Barancik M., Sulova Z. (2013). New insight into p-glycoprotein as a drug target. Anticancer. Agents Med. Chem..

[67] Cole S.P. (2014). Multidrug resistance protein 1 (MRP1, ABCC1), a "multitasking" ATP-binding cassette (ABC) transporter. J. Biol. Chem..

[68] Yakusheva E.N., Titov D.S. (2018). Structure and Function of Multidrug Resistance Protein 1. Biochemistry.

[69] Rosenberg M.F., Mao Q., Holzenburg A., Ford R.C., Deeley R.G., Cole S.P. (2001). The structure of the multidrug resistance protein 1 (MRP1/ABCC1). crystallization and single-particle analysis. J. Biol. Chem..

[70] Wang H., Jin G., Wang H., Liu G., Qian J., Jin L., Wei Q., Shen H., Huang W., Lu D. (2009). Genetic susceptibility of lung cancer associated with common variants in the 3’ untranslated regions of the adenosine triphosphate-binding cassette B1 (ABCB1) and ABCC1 candidate transporter genes for carcinogen export. Cancer.

[71] Low F.G., Shabir K., Brown J.E., Bill R.M., Rothnie A.J. (2020). Roles of ABCC1 and ABCC4 in Proliferation and Migration of Breast Cancer Cell Lines. Int. J. Mol. Sci..

[72] Behfarjam F., Rostamzadeh J., Zarei M.A., Nikkhoo B. (2015). Association of Two Polymorphic Codons in P53 and ABCC1 Promoter with Prostate Cancer. Iran. J. Biotechnol..

[73] Emmanouilidi A., Casari I., Gokcen Akkaya B., Maffucci T., Furic L., Guffanti F., Broggini M., Chen X., Maxuitenko Y.Y., Keeton A.B. (2020). Inhibition of the Lysophosphatidylinositol Transporter ABCC1 Reduces Prostate Cancer Cell Growth and Sensitizes to Chemotherapy. Cancers.

[74] Allikmets R., Gerrard B., Hutchinson A., Dean M. (1996). Characterization of the human ABC superfamily: Isolation and mapping of 21 new genes using the expressed sequence tags database. Hum. Mol. Genet..

[75] Oguri T., Achiwa H., Sato S., Bessho Y., Takano Y., Miyazaki M., Muramatsu H., Maeda H., Niimi T., Ueda R. (2006). The determinants of sensitivity and acquired resistance to gemcitabine differ in non-small cell lung cancer: A role of ABCC5 in gemcitabine sensitivity. Mol. Cancer Ther..

[76] Nedeljkovic M., Tanic N., Prvanovic M., Milovanovic Z., Tanic N. (2021). Friend or foe: ABCG2, ABCC1 and ABCB1 expression in triple-negative breast cancer. Breast Cancer..

[77] Da Silva V.A., Da Silva K.A.E.P., Delou J.M.A., Da Fonseca L.M., Lopes A.G., Capella M.A.M. (2014). Modulation of ABCC1 and ABCG2 proteins by ouabain in human breast cancer cells. Anticancer Res..

[78] Issa M.E., Hall S.R., Dupuis S.N., Graham C.L., Jakeman D.L., Goralski K.B. (2014). Jadomycins are cytotoxic to ABCB1-, ABCC1-, and ABCG2-overexpressing MCF7 breast cancer cells. Anticancer Drugs.

[79] Chen J., Wang Z., Gao S., Wu K., Bai F., Zhang Q., Wang H., Ye Q., Xu F., Sun H. (2021). Human drug efflux transporter ABCC5 confers acquired resistance to pemetrexed in breast cancer. Cancer Cell. Int..

[80] Mourskaia A.A., Amir E., Dong Z., Tiedemann K., Cory S., Omeroglu A., Bertos N., Ouellet V., Clemons M., Scheffer G.L. (2012). ABCC5 supports osteoclast formation and promotes breast cancer metastasis to bone. Breast Cancer Res..

[81] D’Andrea F.P. (2012). Intrinsic radiation resistance of mesenchymal cancer stem cells and implications for treatment response in a murine sarcoma model. Dan. Med. J..

[82] Choi H.J., Jhe Y.L., Kim J., Lim J.Y., Lee J.E., Shin M.K., Cheong J.H. (2020). FoxM1-dependent and fatty acid oxidation-mediated ROS modulation is a cell-intrinsic drug resistance mechanism in cancer stem-like cells. Redox Biol..

[83] Rochat B. (2009). Importance of influx and efflux systems and xenobiotic metabolizing enzymes in intratumoral disposition of anticancer agents. Curr. Cancer Drug. Targets..

[84] Viswanathan S., Parida S., Lingipilli B.T., Krishnan R., Podipireddy D.R., Muniraj N. (2023). Role of Gut Microbiota in Breast Cancer and Drug Resistance. Pathogens.

[85] Terrisse S., Derosa L., Iebba V., Ghiringhelli F., Vaz-Luis I., Kroemer G., Fidelle M., Christodoulidis S., Segata N., Thomas A.M. (2021). Intestinal microbiota influences clinical outcome and side effects of early breast cancer treatment. Cell. Death Differ..

[86] Hou M.F., Ou-Yang F., Li C.L., Chen F.M., Chuang C.H., Kan J.Y., Wu C.C., Shih S.L., Shiau J.P., Kao L.C. (2021). Comprehensive profiles and diagnostic value of menopausal-specific gut microbiota in premenopausal breast cancer. Exp. Mol. Med..

[87] Pricci M., Girardi B., Giorgio F., Losurdo G., Ierardi E., Di Leo A. (2020). Curcumin and Colorectal Cancer: From Basic to Clinical Evidences. Int. J. Mol. Sci..

[88] Pluta R., Januszewski S., Ulamek-Koziol M. (2020). Mutual Two-Way Interactions of Curcumin and Gut Microbiota. Int. J. Mol. Sci..

[89] McFadden R.M., Larmonier C.B., Shehab K.W., Midura-Kiela M., Ramalingam R., Harrison C.A., Besselsen D.G., Chase J.H., Caporaso J.G., Jobin C. (2015). The Role of Curcumin in Modulating Colonic Microbiota During Colitis and Colon Cancer Prevention. Inflamm. Bowel Dis..

